# Associations between Stereotype Awareness, Childhood Trauma and Psychopathology: A Study in People with Psychosis, Their Siblings and Controls

**DOI:** 10.1371/journal.pone.0117386

**Published:** 2015-02-23

**Authors:** Catherine van Zelst, Martine van Nierop, Daniëlla S. van Dam, Agna A. Bartels-Velthuis, Philippe Delespaul, GROUP-investigators

**Affiliations:** 1 Maastricht University Medical Centre, Department of Psychiatry and Psychology, EURON, Maastricht, The Netherlands; 2 Academic Medical Centre, University of Amsterdam, Department of Psychiatry, Amsterdam, The Netherlands; 3 University of Groningen, University Medical Center Groningen, University Center for Psychiatry, Groningen, The Netherlands; 4 Mondriaan Mental Health Trust, South Limburg, Maastricht/Heerlen, The Netherlands; The University of Queensland, AUSTRALIA

## Abstract

**Introduction:**

Stereotype awareness—or an individual’s perception of the degree to which negative beliefs or stereotypes are held by the public—is an important factor mediating public stigma, self-stigma and their negative consequences. Research is required to assess how individuals become more sensitive to perceive stereotypes, pointing the way to therapeutic options to reduce its negative effects and increase stigma resilience. Because perception and interpretation can be guided by belief systems, and childhood trauma (CT) is reported to impact such beliefs, CT is explored in relation to stereotype awareness (SA) in persons with psychosis, their siblings and controls.

**Method:**

Data from the GROUP project (Genetic Risk and Outcome of Psychosis) were analyzed. SA was measured by devaluation scales which assess a respondent’s perception of the degree to which stereotypes about people with mental illness and about their families are held by the public. CT was measured using the Childhood Trauma Questionnaire (short form).

**Results:**

In patients, symptoms of disorganization and emotional distress were associated with SA about people with mental illness. In siblings, schizotypal features were associated with both types of SA (more schizotypy = more SA). In both patients and siblings, CT was associated with both types of SA (more CT = more SA), independent of symptoms (patients) or schizotypy (siblings).

**Conclusion:**

CT in people with psychosis and their siblings may sensitize to SA. Thus, CT may not only impact on risk for illness onset, it may also increase SA associated with mental illness, potentially interfering with the recovery process. CT-induced SA may indicate a heightened sensitivity to threat, which may also impact psychopathology.

## Introduction

Stigmatized individuals possess or are perceived to possess an attribute conveying a devalued social identity within a social context [1]. Both experience of public stigma, indicating negative attitudes and discriminatory behavior toward an individual or group with such (perceived) attributes, and childhood trauma (CT), represent adversities, which can be part of the lives of people with psychosis [2,3]. An important factor in the experience of stigma is one’s perception of stereotypes. Everyone can perceive stereotypes and become target of stigmatization. In the present study, we look at stereotype awareness (SA) in three groups: people with psychosis spectrum disorder, their siblings, and controls. In these groups, we study two domains of stereotypes: stereotypes about patients with mental illness and stereotypes about their families [4]. The scales used assess a respondent’s perception of what the public (“most other people”) believes about patients with a mental illness (DCS) and about their families (DCFS). The DCS informs about the respondent’s perceptions of the public’s view on ‘psychiatric patients’. The DCFS informs about the person’s perceptions of public views on patients’ families, including the patient member(s) of the family and regardless of the psychological health of the other family members (e.g. parents and siblings).

Since both types of SA and CT may influence the formation of dysfunctional beliefs about the self and one’s surroundings, exploring these phenomena can be useful to increase knowledge on how to optimize interventions that increase resilience.

### Childhood trauma and psychosis

A meta-analysis by Varese and colleagues [3] suggests a consistent association between childhood adversity or trauma and psychotic outcomes. They found adversity to be significantly associated with an increased risk for psychosis (OR = 2.78). Heins and colleagues [5] found that the association between CT and psychosis is apparent across vulnerable groups and people with different expression of illness, which suggests a true association rather than reporting bias, reverse causality, or passive gene-environment correlation [5]. Van Dam and colleagues [6] replicated these findings. Results of their study point to trauma as a contributing factor to a shared vulnerability for psychotic and depressive symptoms [6].

**Fig 1:**
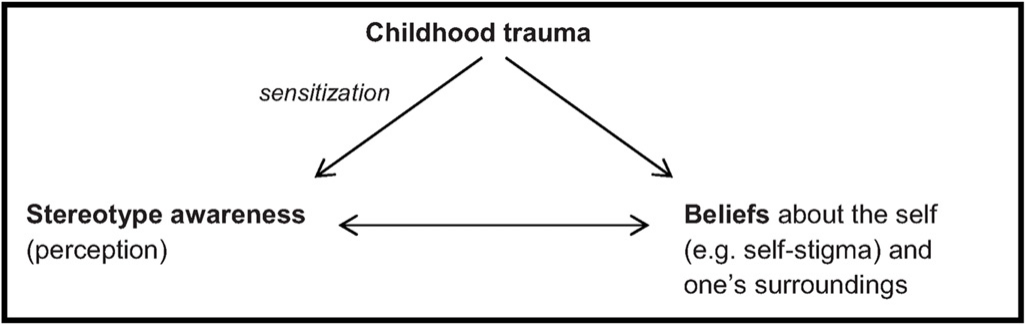
Hypothetical model of associations between childhood trauma, stereotype awareness and beliefs. We hypothesize that CT may sensitize the individual to perceive stereotypes in society (SA). Earlier research has demonstrated that CT impacts on beliefs. We also expect SA to interact with these beliefs.

CT has been found to impact negatively on symptomatic and functional outcomes in psychosis [7]. CT may influence the formation of maladaptive schemas, which may lead to a tendency to mistrust people and withdraw from social situations, as well as a vulnerability to perceive stereotypes and experience stigma. This vulnerability can lead to more traumatic experiences based on previous, present or anticipated trauma (retraumatization). We hypothesize that CT may sensitize the individual to perceive stereotypes in society (SA). Earlier research has demonstrated that CT impacts on beliefs. We also expect SA to interact with these beliefs (Fig. 1).

### The experience of stigma, stereotype awareness and dysfunctional beliefs

Both CT and psychosis are linked to stereotypes, which in turn represent an important component in the development of stigma experiences [8]. Stereotypes or “qualities perceived to be associated with particular groups or categories of people” [9], play a role in both public stigma and self-stigma [10]. Link and colleagues [11] described in their ‘modified labeling theory’ that perceptions of devaluation or discrimination are the first step in a labeling process that influences people when societal stereotypes (once labeled) are applied upon themselves [11]. Corrigan, Watson & Barr [12] defined Link’s process of perceived discrimination as *stereotype awareness* (SA): “the person is aware of the general negative beliefs about mental illness held by one’s culture” (p.876). The experience of stigma may be preceded by or co-occur with SA. Since beliefs about the self, others and the world are important in self-stigma, awareness of stereotypes may play an important role in this development. For example, SA can influence the formation of dysfunctional beliefs. On the other hand, beliefs about the self, others and the world may also be influenced by trauma in childhood. Social marginalization, difficult or traumatic experiences or unsupportive family environments can contribute to the development of maladaptive schemas of the self and the world. The development of maladaptive schemas, which can also be fuelled by chronic stress, can contribute to treatment resistance and a vulnerability to relapse [13].

Society’s attitude to mental disabilities, which has been “ambivalent and marginalizing at best and shunning at worst”, contributes to the impact on the family of a child with a serious mental illness [14].

CT increases vulnerability, possibly through the mechanisms of SA that results in depreciating beliefs about self and others (e.g. “I am worthless” or “Others will reject me”).

To our knowledge, research combining CT, stigma and psychosis is scarce. In one study, the relationships between trauma history, trait anger and stigma were examined [15]. Outcalt and Lysaker reported that stigma experiences can be affected by past experiences of sexual trauma in people with a diagnosis of schizophrenia [15]. Comparison of groups with and without a history of sexual trauma revealed that those who experienced sexual trauma reported more discrimination experience, alienation, and social withdrawal five months later. These results suggest that trauma history may lead to an increased vulnerability to stigmatizing beliefs. More knowledge about determinants of SA may improve treatment and educational interventions aimed at reducing negative consequences of stigma, in patients, family members and possibly others in the broad society.

Studying CT in the context of SA is important, because it may shed light on targets in interventions that aim to decrease the negative impact of CT and/or stigma. The present study explores whether SA and CT are associated, possibly indicating that CT may sensitize individuals for SA and stigma experiences. We hypothesized that CT may increase the salience for negative stereotypes held by the public. Therefore CT may be associated with SA (more CT = more SA).

### Stigma in siblings

Research suggests that health care services are inadequate in meeting the needs for support of siblings of individuals with severe mental illness [16].

When a person experiences a psychotic episode, all family members are affected, but different family roles may evoke different emotional reactions [17]. Parents and siblings can experience stigma themselves. Parent insight into the mental illness of their child increases parent burden because it increases parent self-stigma [18].

In a study among siblings of patients with schizophrenia, participants described their concerns about the impact of a family history of psychiatric illness, the fear of becoming mentally ill as well, and reflections about “bad genes” [17]. Individuals and families dealing with mental disabilities tend to become isolated and feel ashamed [14]. One of the reasons for this may be that they can experience stigma. First, they can experience stigma caused by being associated with a person who is the target of stigmatization: courtesy stigma [19]. Furthermore, being closely affiliated to a stigmatized individual, family members may develop affiliate stigma by which they may feel unhappy and helpless about their affiliation with the stigmatized individual and perceive a negative influence on themselves [20]. Siblings experience emotional, practical and social consequences as a result of their brother or sister developing psychosis [21].

Because patients and siblings may share culture-driven notions of stereotypes, and may to a certain degree be exposed to the same trauma-prone and psychosis-inducing environments, we study the link between CT and SA in people with psychosis and in their siblings. A sample of healthy controls was used as a reference group. Moreover, investigating samples with different (expressed) vulnerability for psychosis may result in increased knowledge of underlying common mechanisms with respect to stigma, stereotype awareness and CT. In a previous study [22], stereotype awareness was found to be associated with psychopathology in patients with psychosis. Lysaker and colleagues found that greater initial stigma predicted greater emotional discomfort at follow-up. Positive symptoms may make some persons with a diagnosis of schizophrenia more vulnerable to ongoing stigma experience [23]. Therefore, in the current study, psychopathology was included as a covariate in the analyses.

We expected a dose-response relationship with the strongest association between CT and SA in patients.

## Methods

Data pertain to the first and second interview wave of the Genetic Risk and Outcome of Psychosis (GROUP) study, an ongoing longitudinal multicenter study in Europe. In selected representative geographical areas of the Netherlands and (the Dutch speaking part of) Belgium, patients were identified through clinicians working in regional psychosis care facilities or academic centers. Eligible patients presenting consecutively at these services either as outpatients or inpatients were recruited for the study. Their siblings were contacted. Controls were selected through a system of random mailings to addresses in the catchment areas of the cases. For a detailed description of objectives, sample characteristics, recruitment and assessment methods, see Korver and colleagues [24].

### Ethics statement

Persons identified as potentially eligible and deemed capable of providing informed consent by their clinician were given detailed explanation of the study procedures and were asked for written informed consent for detailed assessment and for contacting their first-degree family members (brothers, sisters, parents). Written informed consent was also obtained from the next of kin, caretakers, or guardians of those aged 16–17 years. Before written informed consent was obtained, persons had the opportunity to reflect on and ask questions about participation. They could talk about the study with an independent physician who was not involved in the study. All potential participants who declined to participate or otherwise did not participate were eligible for treatment (if applicable) and were not disadvantaged in any way in case of non-participation. The study protocol was approved by the Ethical Review Board of the University Medical Centre Utrecht.

### Subjects

Inclusion criteria were: age range of 16–50 years at baseline, good command of the Dutch language, able and willing to give written informed consent and, in patients, a clinical diagnosis of non-affective psychotic disorder according to the Diagnostic and Statistical Manual of Mental Disorders, fourth edition (DSM-IV) [25]. Furthermore, controls did not have a lifetime psychotic disorder or a first-degree family member with a lifetime psychotic disorder [24]. Only subjects who filled in more than 70% of the items of the SA-scales (see “scales”) were included.

### Scales

**Dependent variable**. *Stereotype awareness* was measured using the Devaluation of Consumers Scale (DCS, 8 items) and the Devaluation of Consumers Families Scale (DCFS, 7 items) [4]. These scales assess a respondent’s perception of what the public (“most other people”) believes about patients with a mental illness (DCS) and their families (DCFS). Using the DCFS next to the DCS may add valuable information about SA in a different context, namely that of the family. More specifically, the DCFS consists of items that enable estimating the extent to which one believes that most people devalue families that include one or more persons with serious mental illness [4]. As such, it taps into the level of contagion, or how stigma may spread, across individuals who are close to the object of stigma.

Examples of DSC items are “Most people would not accept a person who once had a serious mental illness as a close friend” and “Most people will not hire a person who once had a serious mental illness if he or she is qualified for the job”. Examples of DCFS items are “Most people in my community would rather not be friends with families that have a relative who is mentally ill living with them” and—more related to parent-child relationships—“Most people believe that parents of children with a mental illness are not as responsible and caring as other parents”.

All items are rated on 4-point Likert Scales: Strongly disagree (= 1), Disagree, Agree, Strongly agree (= 4). In the statistical analyses, the average item scores were used as overall scores. The higher the score on the DCS or DCFS, the more a person is aware of the general negative beliefs about mental illness held by one’s culture.

Struening and colleagues [4] reported an internal consistency reliability coefficient of 0.82 for the DCS in two samples of caregivers, and coefficients of 0.71 and 0.77 for the DCFS in these samples. Another study into caregivers’ perception of stigma reported a Cronbach’s α for the DCFS of 0.80 [26].

**Independent variables**. *Childhood trauma* was assessed with Dutch version of the 25-item Childhood Trauma Questionnaire—Short Form [27]. Items are rated on 5-point Likert scales (1 = never true, 5 = very often true). The scale rates sexual abuse (sexual contact or conduct between a child and an adult or older person), physical abuse (bodily assaults that posed a risk or resulted in injury), emotional abuse (verbal assaults on a child’s sense of worth or well-being or any humiliating or demeaning behavior directed toward a child by an adult or older person), physical neglect (the failure of caretakers to provide basic physical needs) and emotional neglect (the failure to meet the child’s basic emotional and psychological needs). The CTQ-SF has adequate reliability and content coverage [27]. In the statistical analyses, the individual’s CTQ-SF score was used, representing the mean of the overall scores of the five clusters (sexual, physical and emotional abuse, and physical and emotional neglect).

*Psychopathology* was assessed using the Positive and Negative Syndrome Scale [28] [PANSS; 20], which measured symptom intensity in the two weeks before the interview. Items are rated on a 7-point ordinal scale. For the purpose of the analyses, data were summarized using an empirical model with five factors: positive symptoms, negative symptoms, disorganization, excitement and emotional distress [29]. Psychotic experiences display a dimensional distribution in the general population [30]. Since the PANSS is not sensitive in subjects without psychotic disorder (controls and siblings), we assessed “schizotypy” in these groups with the Structured Interview for Schizotypy—Revised (SIS-R). The SIS-R was developed for assessing subtle schizotypal features in non-psychotic relatives of patients with schizophrenia [31], indexing the psychometric expression of vulnerability for psychotic disorder. For the current analysis, the overall score of the SIS-R was used.

### Statistical analyses

Analyses were done on data release 3.02 of the GROUP study. Since the CTQ-SF was administered during the first wave in the region of Maastricht and during the second wave in the regions of Amsterdam, Groningen and Utrecht, we used data of both waves. Eligible patients were identified in three subsamples (patients, siblings, controls). Statistical analyses were done with STATA 11.2 [32]. Data were analyzed using multilevel linear (ML) regression models with family (nesting variable for patients and siblings) as grouping variable. The ML regression analyses were computed with stereotype awareness (DCS or DCFS) as dependent variable. In the first model, we analyzed the association between SA (DCS or DCFS) and CT (CTQ-SF). “Patients” and “siblings” were added as dummy variables indicating the patient or sibling group, respectively. Controls served as reference category. Interactions of “patients” and CT and “siblings” and CT were added, and sex, age and ethnicity (0 = white, 1 = non-white) were included as *a priori* covariates. In further analyses, we studied the association between psychopathology (PANSS factor scores in patients and SIS-R scores in siblings and controls) and SA. As the impact of CT on SA will likely differ as a function of illness (patient) and/or vulnerability for illness (siblings), interactions between CT and GROUP were added in the models of SA.

## Results

### Subjects

641 patients completed the DCS and 638 the DCFS. Summary scores of these two scales were moderately correlated (r = 0.58). 707 siblings completed the DCS and 704 the DCFS (correlation r = 0.62). 415 controls completed the DCS and 413 the DCFS (correlation r = 0.63) (Table 1). Comparing mean scores of patients, siblings and controls with oneway-ANOVA revealed that the three groups did not differ significantly in SA about patients (DCS: F = 2.69, p = 0.0682), whereas they did differ in the mean SA about families (DCFS: F = 13.39; p = 0.000). Table 1 summarizes demographics, CTQ-scores (mean scores of the five CTQ-clusters), psychopathology and SA scores.

**Table 1:** Demographics, psychopathology and stereotype awareness.

Variable	Patients (N = 641)	Siblings (N = 707)	Controls (N = 415)
Proportion male	75%	45%	45%
Age (years) (SD; range)	30.2 (7.1; 18–53)	30.4 (7.6; 17–53)	33.9 (10.4; 18–53)
Diagnosis	96% Schizophrenia or other psychotic disorder	79% None	85% None
	2% Depressive disorder	17% Depressive disorder	13% Depressive disorder
	1% Common mental disorder	4% Common mental disorder	2% Common mental disorder
	1% Unknown		
Duration of illness (years) (SD; range)	7.7 (4.2; 2.0–43.1)		
Ethnicity	84% white ethnicity	88% white ethnicity	93% white ethnicity
	9% mixed ethnic group	8% mixed ethnic group	5% mixed ethnic group
	2% Moroccan	1% Moroccan	1% Moroccan
	2% Surinamese	1% Surinamese	1% other ethnicity
	2% Turkish	1% Turkish	
	1% other ethnicity	1% other ethnicity	
CTQ-SF overall score (SD; range)	1.6 (0.5; 1–3.8)	1.4 (0.4; 1–4.3)	1.3 (0.3; 1–3.1)
PANSS Positive symptoms (SD; range)	11.7 (5.8; 3–39)		
PANSS Negative symptoms (SD; range)	12.5 (5.3; 4–38)		
PANSS Disorganization (SD; range)	14.4 (5.3; 10–46)		
PANSS Emotional distress (SD; range)	13.4 (5.0; 8–33)		
PANSS Excitement	10.7 (3.3; 8–29)		
SIS-R overall score (SD; range		0.3 (0.2; 0–1.4)	0.3 (0.2;0–1)
DCS overall score (SD; range	2.5 (0.5; 1–4)	2.5 (0.4; 1–4)	2.4 (0.4; 1–3.6)
DCFS overall score (SD; range	2.1 (0.5; 1–4)	2.0 (0.5; 1–3.4)	2.0 (0.5; 1–4)

*CTQ-SF = Childhood Trauma Questionnaire—short form*, *PANSS = Positive and Negative Syndrome Scale*, *SIS-R = Structured Interview for Schizotypy—Revised*, *DCS = Devaluation of Consumers Scale*, *DCFS = Devaluation of Consumers Families Scale*.

### Childhood trauma and stereotype awareness

**Fig 2:**
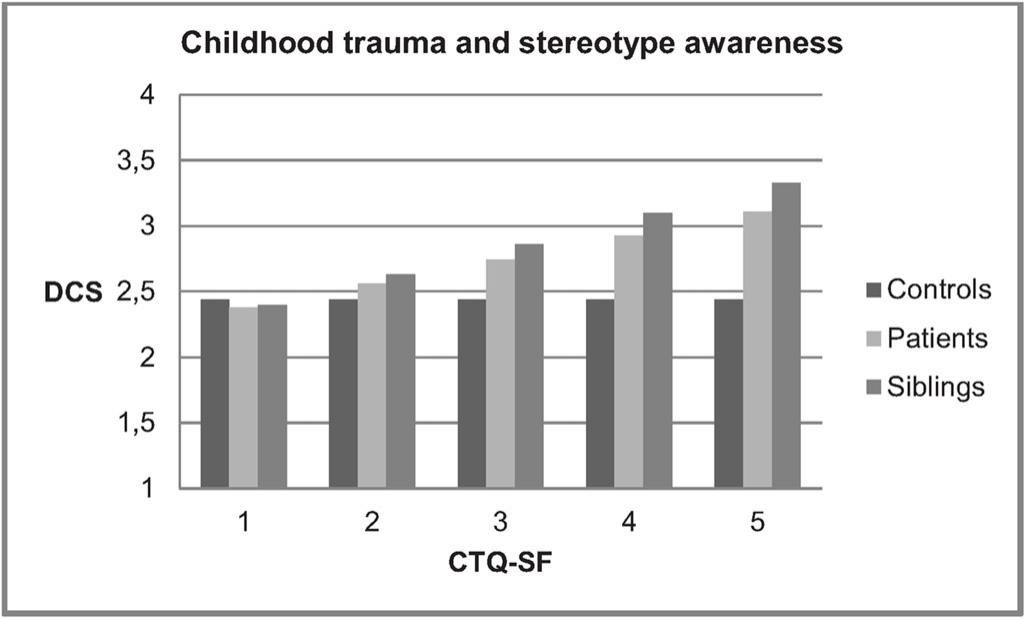
Childhood trauma (CTQ-SF) and stereotype awareness (DCS) in patients, siblings and controls. Example: Results for 30-year-old men of white ethnicity. CTQ-SF = Childhood Trauma Questionnaire—Short Form. DCS = Devaluation of Consumers Scale. Presented are scores on DCS associated with CTQ-SF scores (maximum CTQ-SF range of 1 (= all items rated as “never true”) until 5 (= all items rated as “very often true”).

**Fig 3:**
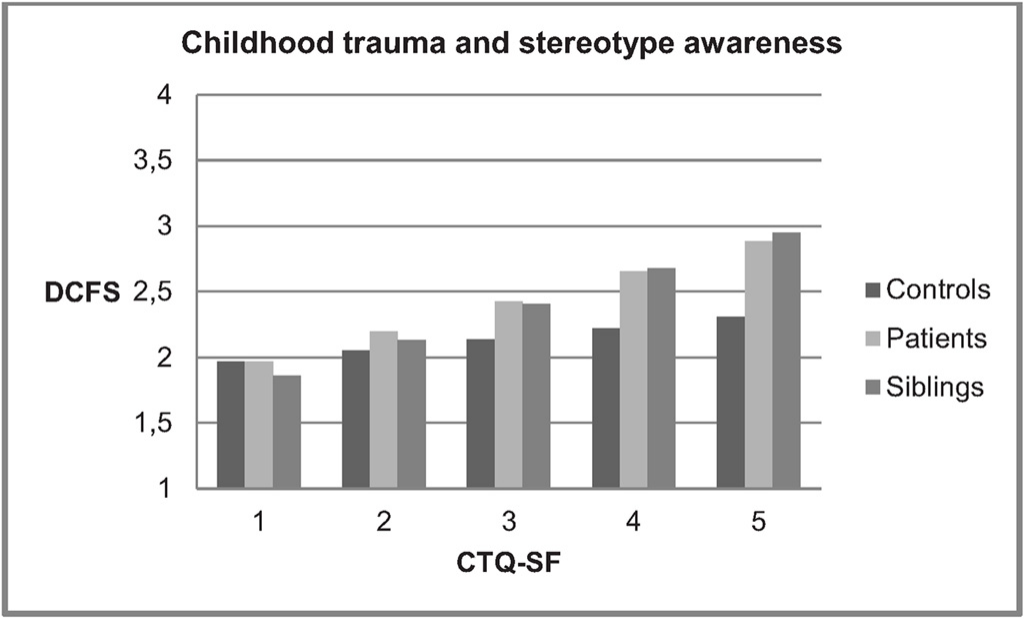
Childhood trauma (CTQ-SF) and stereotype awareness (DCFS) in patients, siblings and controls. Example: Results for 30-year-old men of white ethnicity. CTQ-SF = Childhood Trauma Questionnaire—Short Form. DCFS = Devaluation of Consumers Families Scale. Presented are scores on DCFS associated with CTQ-SF scores (maximum CTQ-SF range of 1 (= all items rated as “never true”) until 5 (= all items rated as “very often true”).

All regression models were adjusted for sex, age and ethnicity. In the first model (Table 2), group membership was associated with DCS, siblings and patients scoring higher than controls, as well as with DCFS, siblings scoring higher than controls. There was no association between CT and SA, but in this model a significant interaction between group and CT was apparent. Figs. 2 and 3 show associations between CT and DCS for these first two models.

**Table 2:** Results of multilevel regression analysis on stereotype awareness in patients, siblings and controls (reference category).

	DCS		DCFS	
	β	p	β	p
Group siblings	-0.29	0.015*	-0.30	0.012*
Group patients	-0.25	0.032*	-0.14	0.219
CTQ-SF	0.00	0.991	0.08	0.229
Group siblings * CTQ-SF	0.37	0.004*	0.28	0.026*
Group patients * CTQ-SF	0.33	0.020*	0.25	0.074

*Results adjusted for sex, age and ethnicity*

*CTQ-SF = Childhood Trauma Questionnaire—short form, DCS = Devaluation of Consumers Scale, DCFS = Devaluation of Consumers Families Scale*.

Given significant interaction, stratified analyses were conducted for each group. In the patients, there was a positive association between CT and SA for both DCS and DCFS (more CT = more SA), independent of symptoms. Furthermore, two PANSS factors were associated with DCS (not DCFS): PANSS disorganization and PANSS emotional distress, independent of CT. PANSS disorganization was negatively associated with DCS and PANSS emotional distress positively (Table 3).

**Table 3:** Results of multilevel linear regression analysis on stereotype awareness in patients.

Patients	DCS		DCFS	
	β	p	β	p
CTQ-SF	0.17	<0.001*	0.19	<0.001*
PANSS Positive symptoms	0.05	0.487	-0.01	0.895
PANSS Negative symptoms	0.04	0.379	0.08	0.093
PANSS Disorganization	-0.15	0.011*	-0.03	0.571
PANSS Emotional distress	0.14	0.017*	0.11	0.070
PANSS Excitement	0.08	0.182	0.06	0.291

*Results adjusted for sex, age and ethnicity*

*DCS = Devaluation of Consumers Scale; DCFS = Devaluation of Consumers Families Scale; CTQ-SF = Childhood Trauma Questionnaire—short form; PANSS = Positive and Negative Syndrome Scale.*

In the siblings, there was a positive association between CT and SA (both DCS and DCFS), independent of SIS-R score. SIS-R overall score was also significantly associated with SA, independent of CT (more SIS-R symptoms, more SA (both DCS and DCFS)) (Table 4).

Models including both CT and SIS-R overall score as independent variables were not significant for controls. However, elimination of CT from the model revealed a significant positive association between SIS-R overall score and SA (in the DCS as well as the DCFS model).

**Table 4:** Results of multilevel linear regression analyses on stereotype awareness in siblings.

Siblings	DCS		DCFS	
	β	p	β	p
CTQ-SF	0.16	<0.001*	0.16	<0.001*
SIS-R overall	0.13	0.001*	0.21	<0.001*

*Results adjusted for sex, age and ethnicity*

*DCS = Devaluation of Consumers Scale; DCFS = Devaluation of Consumers Families Scale; CTQ-SF = Childhood Trauma Questionnaire—short form; SIS-R = Structured Interview for Schizotypy—Revised.*

## Discussion

The aim of this study was to explore SA, a factor representing stigma vulnerability, which can precede or co-occur with self-stigma, and assess its relation to childhood trauma (CT). Awareness of stereotypes was lower than expected.

In both patients and their siblings, CT was associated significantly with the awareness of stereotypes about patients and their families. In interpreting the results, it should be noticed that patients and siblings may share environmental factors that can include or induce stigma experiences. Furthermore, while patients are possible targets of stereotypes and stigmatization, siblings may also be exposed (e.g. courtesy stigma, or vicariously learning or experiencing that their siblings may be or are in fact stigmatized because of mental illness). Besides, one should note that the DCS and DCFS cover different domains of SA, not only because of the different groups that are object of SA (patients and families respectively) but also given the more person-centred approach of the DCS as opposed to the systemic approach of the DCFS (one can consider the whole family and its dynamics regardless of the health of its individual members).

The fact that CT was stronger associated with SA in siblings compared to patients may represent the effect of (medication-induced) indifference as part of negative symptoms (withdrawal, blunted affect, apathy) in patients compared to siblings. Qualitative research may further inform on how SA is experienced by patients and siblings respectively.

In patients, SA (DCS-scale) was also associated, independently of CT, with symptoms of disorganization (more disorganization = less SA) and emotional distress (more emotional distress = more SA). However, replication is needed in order to inform interventions on coping with symptoms as a possible contributing factor to stigma management. In siblings, SA was additionally and independently associated with schizotypal features (more schizotypy = more SA). In controls, an association was found between schizotypal features and SA, but not between CT and SA. Being aware of stereotypes is a prerequisite to experience stigma, and CT experiences may bring about sensitization to SA. For treatment and psycho-educational purposes, it would be helpful to know how CT is linked to SA.

People with a history of trauma may find it difficult to accurately appraise the self and the world. Many reports have documented how trauma reshapes the self-perception of the survivor, resulting in decreased self-esteem, shame, stigma, and guilt [33]. A potential underlying mechanism mediating the impact of trauma and leading to negative and stigmatizing beliefs about the self, is that traumatized persons can be prone to unconsciously scanning the environment for threat [34], which may serve as a confirmation of negative self-beliefs [15]. This may represent a bias resulting from rejection sensitivity. Another mechanism representing such bias concerns difficulty disengaging from trauma [35].

People differ in their readiness to perceive and react to rejection [36]. Rejection sensitivity develops through repeated and prolonged experiences of rejection. This sensitivity results in altered perception and behavior in situations where rejection is imminent. Rejection sensitivity is assumed to become a trait characteristic of the individual, but it is more strongly activated in ‘threatening’ situations and therefore also state dependent [37].

Sensitization of an individual to detect stereotypes may trigger a range of biases, for example “negative underlying assumptions” (NUAs) [38,39]. The development of negative underlying assumptions about, for example, performance evaluation and approval by others after experiencing trauma may result in negative feelings such as shame. A study in undergraduate students indicated that individuals who are able to maintain low levels of negative underlying assumptions after trauma may be resilient to the potentially shaming effects of negative feedback [38]. A low level of NUAs may be accompanied by stigma resilience as well. Additionally, high group value, when people with mental illness hold their group in high regard, and low perceived legitimacy of discrimination, when people reject stigma as unfair, may contribute to resilience to stigma [40].

CT may induce behavioral sensitization to adult stress in individuals with increased psychosis liability [41]. In this sensitization process people may become, by previous exposure to adversity or stress, more sensitive or responsive rather than more resistant to the later occurrence of stress [41,42]. In addition, appraisal of experiences—influenced by reasoning and attributional biases, dysfunctional schemas of self and world, and isolation and adverse environments—influences the development of, amongst others, positive symptoms [13,43].

In siblings, both stigma directed at people with mental illness as well as stigma directed at their families can be a source of distress. Patients and their siblings may have shared experiences of psychopathology and stigma, associated with earlier environmental exposures that cluster in the family. In future research, information about well controls and their experiences with mental illness in others, a factor that may influence their stereotype awareness, may advance our understanding of processes of stereotype awareness in society. Future research into siblings may inform us on how their vulnerability and resilience with respect to developing psychosis or psychotic symptoms may be influenced by stigma experiences.

### Interventions

Building and reinforcing resilience is important. By remediating the possible consequences of both SA and CT, underlying beliefs about the self, others and the world can be addressed. Interventions aimed at decreasing self-stigma may also increase resilience against the consequences of SA. With respect to decreasing self-stigma (or “internalized stigma”), “narrative enhancement and cognitive therapy” (NECT) has been developed and evaluated [44,45].

The negative impact of stereotyping and stigmatization of persons with mental health problems should also be addressed in society as a whole. People may experience stigma and be aware of stereotypes before onset of disorder or diagnosis [46]. Consequently, both CT and anticipated stigma may delay help-seeking, which can increase the risk of poor outcome. Early detection of mental health complaints, and a focus that incorporates (anticipated) stigma experiences, may thus be useful. Normalizing mental health complaints can be an aim in mental health campaigns. Since SA does not lead to self-stigma in all individuals, it would be interesting to examine the vulnerability and resilience factors influencing the process by which SA may lead to negative, but possibly also positive consequences (e.g. empowerment). Based on this, treatment programmes can be further developed. In a study by Sin and colleagues, “resilience was identified by a number of siblings, who considered that they and their families were completing a difficult journey feeling stronger and more cohesive” [21]. Studying resilience and assisting people in becoming more resilient, e.g. in treatment, are very important.

Educational interventions in schools may decrease stigma experiences (i.e. the negative impact of SA) of adolescents with mental health complaints.

Youths have limited life experience with integration and identity formation. It is likely that, compared with adults, adolescents have a less consolidated identity to protect against, buffer, or neutralize stereotypes and prejudice [47]. Therefore, management of SA and other experiences of stigma should be part of the psychological interventions from the very onset of psychotic experiences. This is of relevance for educational practice as well.

In order to enhance resilience, professionals may carefully establish vulnerabilities and protective factors and decide on the best course of action to improve resilience [48].

Metacognition may be defined as a spectrum of mental activities involving thinking about thinking [49]. A greater metacognitive capacity, better self-esteem, and less negative symptoms are associated with stigma resistance [49]. In a previous study on SA, we found self-esteem to be associated with SA [22]. Mashiach-Eizenberg and colleagues found that self-esteem mediated the association between *internalized stigma* and hope, and hope partially mediated the relationship between self-esteem and quality of life [50]. Results of a study by Hasson-Ohayon and colleagues point to a mediating role of shame-proneness between insight and *self-stigma* [51], suggesting that shame-proneness can be conceptualized as a vulnerability factor for self-stigma experiences. Further investigating the roles of hope, insight and shame in the experience of SA may further contribute to interventions for this type of stigma-related experience as well. Enhancing self-experience is important in recovery and treatment. Experiencing the self as diminished or barren may put one at risk for internalizing public stigma, as one may have no alternative internal experience that can reject stigma [52].

Harvey [53] describes a useful ecological model of psychological trauma and trauma recovery. An ecological model highlights, in acknowledging the multidimensional nature of trauma recovery and the possibility of recovery in the absence of clinical intervention, the construct of resilience, the role of the wider social environment, the contributions of natural supports, and the relevance of community interventions to alter discriminating beliefs.

For individuals who struggle with both CT- and SA-related experiences, addressing these two domains in treatment may be advantageous. Further research may elucidate whether incorporating both domains in treatment and recovery programmes is advisable and for which individuals specifically.

### Limitations

This study was cross-sectional. A large sample size implies that statistically different results are more easily found, while the chance of type 1 errors is increased. For these reasons, replication of the study, and a longitudinal study design, are required.

There were some differences in demographics between patients, siblings and controls. While sex, age and ethnicity were controlled for in statistical analyses, we did not specifically study the possible impact of diagnosis in the control and sibling groups. A subgroup of both siblings (21%) and controls (15%) had a diagnosis of a mental disorder, which may have influenced the results.

Research may benefit from more specific analyses focusing on different types of CT instead of using an overall CT-score. Different types of CT (e.g. abuse vs. neglect, or further specified in different types of abuse and neglect) may be differently associated with SA.

Although this study shows a relationship between CT and SA, it does not inform us on the consequences of SA for the individual. Further research may reveal which factors contribute to stigma resilience and which factors heighten the negative impact of SA. People with psychosis can also experience trauma in other stages of life. Further studies may inform us on whether and to what extent our findings can be generalized to re-victimization in adulthood and to what extent overcoming traumatic experiences may contribute to empowerment. Psychosis in itself is often considered a traumatic experience, and ways to cope with this type of trauma should be addressed in future interventions. Furthermore, attachment theory can be applied in research combining trauma, psychosis and stigma experiences. One of this theory’s advantages is that it maintains our focus on day-to-day circumstances of childhood as well as more obvious, discrete abusive events [54]. Future studies can elaborate on results of SA in siblings.

Some items of the DCFS) appear to tap into child-parent relationships, implicating CT, for example the item on perceived lack of responsibility and caring of parents of children with a mental illness. DCS/DCFS-items can be interpreted differently by patients, siblings and controls, as a function of the particular group they represent in DCS and DCFS terminology. This should be kept in mind when comparing results of DCS or DCFS between groups. While the DCS may be personally relevant to patients, DCFS may hold more personal significance for siblings.

### Conclusion

CT in people with psychosis and their siblings may sensitize to SA. Thus, CT may not only impact on risk for illness onset, it can also increase SA associated with mental illness, potentially interfering with the recovery process. CT-induced SA may indicate a heightened sensitivity to threat, which may also impact psychopathology.
